# The Magnetic Resonance Imaging Pattern of the Lesions Caused by Knee Overuse in the Pediatric Population

**DOI:** 10.3390/medicina58081107

**Published:** 2022-08-16

**Authors:** Goran Djuricic, Djordje Milojkovic, Jovana Mijucic, Sinisa Ducic, Bojan Bukva, Marko Radulovic, Nina Rajovic, Petar Milcanovic, Natasa Milic

**Affiliations:** 1 University Children’s Hospital, Faculty of Medicine, University of Belgrade, 11000 Belgrade, Serbia; 2 Faculty of Medicine, University of Belgrade, 11000 Belgrade, Serbia; 3 Institute of Oncology and Radiology of Serbia, Faculty of Medicine, University of Belgrade, 11000 Belgrade, Serbia; 4 Institute for Medical Statistics and Informatics, Faculty of Medicine, University of Belgrade, 11000 Belgrade, Serbia; 5 Laboratory for Sports Institute of Medical Physiology, Faculty of Medicine, University of Belgrade, 11000 Belgrade, Serbia; 6 Department of Internal Medicine, Division of Nephrology and Hypertension, Mayo Clinic, Rochester, MN 55901, USA

**Keywords:** knee injury, children, magnetic resonance imaging, physical activity, overuse syndrome

## Abstract

*Background and Objectives*: Excessive use of the knee in patients with immature locomotor systems leads to a whole spectrum of morphological changes with possible consequences in adulthood. This study aimed to examine the morphological pattern in magnetic resonance imaging (MRI) that is associated with recurrent pain due to increased physical activity in children. *Materials and Methods:* This was a retrospective study conducted among pediatric patients treated at the University Children’s Hospital in Belgrade in 2018 and 2019. MRI findings of patients who reported recurrent pain in the knee joint during physical activity and who were without any pathological findings on both clinical examination and knee radiographs were included in the study. *Results*: MRI findings of 168 patients (73 boys and 95 girls, mean age 14.07 ± 3.34 years) were assessed. Meniscus and cartilage lesions were the most commonly detected morphological findings: meniscus lesions in 49.4%, cartilage ruptures in 44.6%, and cartilage edema in 26.2% of patients. The medial meniscus was more often injured in girls (*p* = 0.030), while boys were more prone to other joint injuries (*p* = 0.016), re-injury of the same joint (*p* = 0.036), bone bruises (*p* < 0.001), and ligament injuries (*p* = 0.001). In children older than 15 years, tibial plateau cartilage edema (*p* = 0.016), chondromalacia patellae (*p* = 0.005), and retropatellar effusion (*p* = 0.011) were detected more frequently compared to younger children. *Conclusions*: Children reporting recurrent knee pain due to increased physical activity, without any detected pathological findings on clinical examination and knee radiography, may have morphological changes that can be detected on MRI. Timely diagnosis of joint lesions should play a significant role in preventing permanent joint dysfunction in the pediatric population as well as in preventing the development of musculoskeletal diseases in adulthood.

## 1. Introduction

The benefit of frequent physical activity is an important scientific topic, especially due to the increasingly sedentary lifestyle among young people. It is well known that sports encourage proper growth and development in children [1]. On the other hand, due to the low awareness of the risks and consequences associated with excessive physical activity and intense sports, the incidence of injuries in the pediatric population continues to increase, but overuse injuries are still undervalued [2,3,4]. Following both personal and parental ambitions, children are likely to exceed their ability limits [5]. Intense training that is not adapted to children’s growth spurt, age, and biological maturity leads to the damage of joint structures [3,4]. Coaches and parents are usually unaware of the possible extent of the consequences [3,6]. To achieve the best possible results, children often focus on a single discipline, resulting in overuse injuries of the most stressed joints and the accompanying soft tissue [7,8,9].

Injuries of the lower extremities are more common in the pediatric population, and the pathology is specific due to the unique structural physiology of the growing skeleton [3,10]. This is explained by the rapid bone growth associated with a dynamic change in mineral density and disproportion between bone length and musculoskeletal structures [6], hormonal changes, and poor neuromuscular control [11], making younger people more susceptible. With pain being the only symptom, underdiagnosis is a frequent phenomenon in the pediatric population. Children experience pain only after excessive physical activity, and it mostly disappears after proper rest. The recurrence of pain is not followed by any abnormal clinical findings or pathological radiography. If an adequate diagnosis is not performed, these patients may not undergo timely treatment [12].

Magnetic resonance imaging (MRI) is considered the reference standard modality in the radiological diagnosis of joint lesions and the accompanying soft tissue [13]. Due to its high resolution, it offers excellent multi-planar imaging and differentiation of soft tissue, bone marrow, cartilage, muscles, ligaments, and tendons. Furthermore, the absence of ionizing radiation makes MRI the method of choice for diagnostic studies involving children due to their extensive vulnerability to radiation in comparison to adults [14]. Nevertheless, MRI’s use as a diagnostic tool in children is often delayed due to a number of concerns that have been raised concerning potential hazards associated with its use in the pediatric population [15], such as those aiming to define (1) potential physical or psychological harms associated with MRI in the pediatric population and (2) under what circumstances MRI’s use as a diagnostic tool in children meets the minimal risk standard. With the continuation of such dilemmas, determining standard protocols for MRI use can be one of the most difficult tasks. To date, risk assessment in studies on the use of MRI has been inconsistent, ranging from minimal to high risk [15], while studies assessing the severity of morphological changes in joints detected by MRI are still lacking. Thus, in this study, we aimed to examine the morphological clues in MRI that are associated with recurrent pain due to increased physical activity in children and to discuss considerations for using MRI in the pediatric population.

## 2. Materials and Methods

### 2.1. Study Population

This was a retrospective study conducted among pediatric patients treated at the University Children’s Hospital in Belgrade during the years 2018 and 2019. Patients’ knee MRI studies were systematically reviewed after they were obtained from the Picture Archiving and Communication System (PACS). Patients with MRI findings such as acute injuries, malignant or benign tumors, osteomyelitis, juvenile rheumatoid arthritis, and metabolic diseases were excluded from the study. Patients who reported recurrent discomfort, pain, and/or limited mobility in the knee joint during/after physical activity caused by repeated microtrauma without a single, identifiable event responsible for the injury were included in the study and defined as having overuse syndrome [16].

### 2.2. Data Collection and Ethical Approval

For pediatric patients who obtained a knee MRI and entered the study cohort, demographic, clinical, and other radiographic data were obtained. Collected data were de-identified and treated with confidentiality. Informed consent for the use of MRI for diagnostic purposes and its data use was obtained from the parents/guardians of each pediatric patient included in the study. Ethical approval was obtained from the Institutional Review Board (no.: 017-16/14).

### 2.3. Magnetic Resonance Imaging (MRI)

Examinations were performed on MAGNETOM Aera 1.5T using a knee coil, positioned in supination with FOV (field of view) of 16 cm using Ax Int FS, Cor Int FS, Cor T1, Sag Obl Int FS, Sag Obl PD, and Cor Obl PD. The axial sections were parallel to the knee joint line; they included the entire patella and the head of the fibula. The coronal sections were parallel to the femoral condyle’s posterior aspect, including the entire patella and up to 2 cm posterior to the femoral condyle. The sagittal sections were parallel to the medial aspect of the lateral condyle, including both collateral ligaments. All knee MRIs were independently reviewed by a board-certified radiologist (GDJ) with >10 years of experience in musculoskeletal imaging in the pediatric population, blinded to the initial reports.

### 2.4. Statistical Analysis

Numerical data are presented as means with standard deviations or as medians with ranges. Categorical variables are summarized with absolute numbers with percentages. Gender- and age-related differences in the prevalence of specific morphological substrates were assessed using the Chi-square test. In all analyses, the significance level was set at 0.05. Statistical analysis was performed using IBM SPSS statistical software (SPSS for Windows, release 25.0, SPSS, Chicago, IL, USA).

## 3. Results

Out of 382 initially examined MRI studies, a total of 168 who met the criteria for overuse injury were enrolled in the study. There were 73 boys (43.5%) and 95 girls (56.5%). The mean age was 14.07 ± 3.34 years; 44% were under 15 years old, and 56% were 15 years or older (Table 1). All patients asked for medical help after experiencing recurrent pain, mostly in the first three months after the pain occurrence (81.5%). The left knee was slightly more often injured (51.2%) in comparison to the right knee (48.8%). An association between the injured and dominant leg was not established (*p* > 0.05). An earlier injury of another joint was reported in 23.8% of the patients, while 27.4% had a previous injury of the same knee (Table 1).

Pathological findings on MRI were detected in all examined (*n* = 168) pediatric patients with recurrent pain after physical activity (Table 2). Meniscus lesions were detected in almost half of the patients (49.4%), with the medial meniscus affected in 35.1% and the lateral meniscus affected in 22% of patients. Seventy-five patients (44.6%) had cartilage rupture detected on MRI, with the tibial plateau most affected, followed by the femoral condyle and patella. Edema was present in 26.2% of the patients (Table 2).

Boys were more likely than girls to have other joint injuries (32.9% vs. 16.8%, respectively, *p* = 0.016), as well as re-injury of the same joint (35.6% vs. 21.2%, respectively, *p* = 0.036). In addition, a higher frequency of bone bruises (*p* < 0.001) and ligament injuries (*p* = 0.001) was detected among boys. Medial meniscus lesions significantly differed between patients, with girls having more medial lesions than boys (*p* = 0.030) (Table 2). There were no other gender-related differences in morphological substrates detected by MRI (*p* > 0.05).

**Figure 1:**
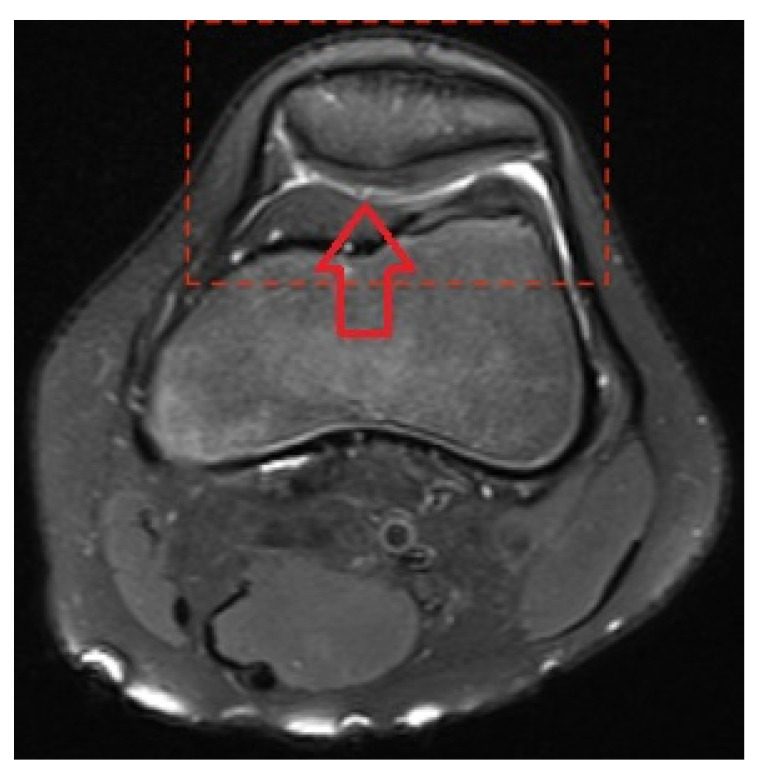
Patellar cartilage rupture.

**Figure 2:**
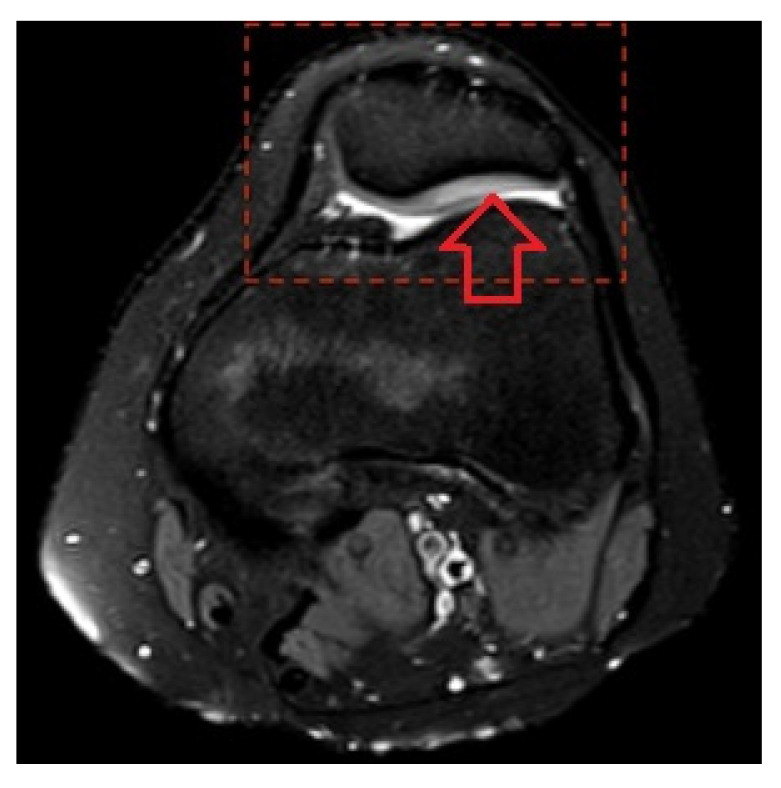
Patellar cartilage edema and retropatellar effusion.

A positive correlation was found between rupture of the patellar cartilage and edema of the femoral condyle cartilage (Figure 1). In contrast, a negative correlation was found between rupture of the femoral condyle cartilage and edema of the patellar cartilage (Figure 2), demonstrating that in patients with rupture, the incidence of edema was decreased. In children older than 15 years, an increasing prevalence of tibial plateau cartilage edema (*p* = 0.016), chondromalacia patellae (*p* = 0.005), and retropatellar effusion (*p* = 0.011) was detected (Table 3). There were no other age-related differences in morphological substrates detected by MRI (*p* > 0.05).

## 4. Discussion

This study showed that lesions caused by knee overuse were detected by MRI in all children who reported recurrent pain in the knee joint during physical activity. In the pediatric population, knee injuries occur due to acute trauma, such as a fall, or excessive use in the field of pre-existing microtraumas [17,18]. It has been shown that morphological substrates accompanied by pain can lead to various orthopedic and rheumatic diseases (such as osteoarthritis) in adulthood [19]. Cartilage lesions (edema and rupture) are the most prevalent cause of pain cited in the literature [20]. According to the results of our research, cartilage rupture was observed in almost half of the patients, with the tibial plateau most affected, followed by the femoral condyle and patella. Cartilage edema was present in 25% of pediatric patients. The most frequent was patellar cartilage edema, while cartilage edema of the femoral condyle and tibial plateau was less common. The study showed an increased incidence of tibial plateau cartilage edema in children older than 15 years. To our knowledge, other researchers have not investigated the individual prevalence of edema and rupture but categorized them as a common category of cartilage damage. Our results show that those two pathological conditions should be separated due to established patterns of different structures’ injuries within the knee joint. Cartilage edema represents a reversible and less harmful pathological condition. As long as the volume remains unaffected, joint functionality stays intact. Conversely, due to its avascular nature, once ruptured, cartilage cannot be regenerated, meaning that functional deficiency is inevitable. In relation to the patellofemoral joint, when rupture of the patellar cartilage is diagnosed, there is corresponding edema of the femoral condyle cartilage, which increases the probability of repeated microtrauma. Seeley et al. monitored the health condition of 122 patients (11–18 years old) for ten years. This study included 46 of 122 patients with a previously diagnosed knee injury and a history of recurrent patellar dislocation. Based on the obtained results, they concluded that cartilage injuries are highly correlated with recurrent microtraumas. Depending on the location of the injury, different functional limitations may occur [20].

Lower extremities are more often injured in the pediatric population due to bone growth and disproportion between bone length and accompanying structures [6,21]. Lambers et al. examined the frequency of lower extremity injuries among different age groups (0–100 years) in a population of 119,815 patients (52% male and 48% female) and concluded that these injuries are commonplace in younger people [22]. In their three-year research, which included male (≤14 years old) and female (≤12 years old) patients, Funahashi et al. [23] examined the incidence of cartilage and meniscus injuries due to recurrent knee injuries and surgical treatment. They included 71 patients, and according to their results, cartilage lesions did not predominate over other structural knee injuries [23]. On the other hand, Ingram et al. concluded that there were no statistically significant differences between genders, and that cartilage was more often injured than accompanying ligaments in a group of 1383 American high school students with knee injuries [24].

One of the most frequently detected lesions in immature knees is meniscus injury. In our study group, meniscus injury was present in half of the pediatric patients. The medial meniscus was more often injured than the lateral. Our results are in line with a survey by Zobel et al., who reported that, in their group of examinees, the medial meniscus was injured more often than the lateral meniscus or anterior crucial ligament [25]. In contrast, Shieh et al., whose survey included 293 patients divided into adolescents (174) and young children (119), showed a higher incidence of lateral meniscus injuries. Additionally, their survey found that injuries were more common in adolescents (61%) than in younger children (39%) [26].

Analysis of the obtained results showed that retropatellar effusion was associated with patellar cartilage edema in every third child. This finding may indicate the importance of cartilage edema as a potential predisposing and/or associated factor with exudation. It is important to highlight that all of the examined patients had no other clinical findings except the pain associated with increased activity. This pain subsides over time but reappears after every knee overuse. In patients whose locomotor system is still growing and developing, knee pain and suspected joint swelling should be noted, and MRI should be indicated for a detailed evaluation of the knee joint morphology [27,28]. MRI can provide information about the cartilage, capsule, and ligaments and allow evaluation of the actual size of the joint lesion. This radiological diagnosis is crucial in the assessment of whether surgical treatment is required [7,29]. Morphological substrates detectable by MRI that correlate with knee pain have been observed in the past. Specifically, the examination of lower extremity injury frequencies in different age groups showed a higher prevalence among younger patients [22]. MRI with superior sensitivity and specificity can detect stress fractures better than both CT and radiographs [30]. The advantages of this imaging method, such as a lack of ionizing radiation, exceptional resolution, and precise distinction of the physis, apophysis, and surrounding soft tissue, make MRI especially advantageous for the pediatric population [6].

In children who perform intense physical activities, the most common symptom is knee pain; however, establishing the right time for diagnostic imaging remains unclear. Children experience pain for a long period of time, yet trainers and physicians fail to recognize the importance and risks of joint damage if the proper diagnosis is not made on time. MRI use opens the possibility of timely and effective prevention of the underlying disease and may reduce the risk of possible further damage, the occurrence of pain, and suffering from deteriorating quality of life of vulnerable pediatric patients. Appropriate risk reduction strategies aimed at well-timed MRI diagnosis in the pediatric population are a necessity, but there is a pressing need for developing and implementing protocols that are reviewed by multidisciplinary teams of experts in the field of radiology and pediatrics in order to benefit children’s health.

The main limitations of this study are that data were obtained retrospectively from medical charts and that patients were recruited from a single center. More detailed orthopedic tests and examinations are required in order to obtain additional results. Another limitation is the actual use of the phrase “overuse injury” or “overuse syndrome”, which still has a vague definition across the literature [31]. Establishing field-wide multicenter and interdisciplinary cooperation with the use of standardized methodological and analytical approaches may allow researchers to gain additional insights into the morphological patterns responsible for pain recurrence in young athletes.

## 5. Conclusions

Children reporting recurrent knee pain due to increased physical activity may have morphological changes that can be detected on MRI. Meniscus lesions and cartilage rupture were the most frequent morphological findings, observed in almost half of the examined patients. Timely diagnosis of joint lesions should play a significant role in preventing permanent joint dysfunction in the pediatric population as well as in preventing the development of musculoskeletal diseases in adulthood. The results of this study support the use of MRI as a diagnostic procedure during the clinical follow-up of children with recurrent knee joint pain.

## Figures and Tables

**Table 1:** Study population.

	*n* (%)
**Gender**	
Male	73 (43.5)
Female	95 (56.5)
**Age**	
<15 years	74 (44.0)
≥15 years	94 (56.0)
**Knee**	
Left	86 (51.2)
Right	82 (48.8)
**Previous injury**	
Same joint	46 (27.4)
Other joint	40 (23.8)

**Table 2:** Morphological substrates detected on MRI in pediatric patients with knee overuse syndrome, overall and gender-related differences.

	Total, *n* (%)	Boys, *n* (%)	Girls, *n* (%)
**Cartilage edema**	45 (26.8)	19 (26.0)	26 (27.4)
Femoral condyle	8 (4.8)	5 (6.8)	3 (3.2)
Tibial plateau	7 (4.2)	3 (4.1)	4 (4.2)
Patella	32 (19.0)	13 (17.8)	19 (20.0)
**Cartilage rupture**	75 (44.6)	33 (45.2)	42 (44.2)
Patella	10 (6.0)	5 (6.8)	5 (5.3)
Femoral condyle	19 (11.3)	8 (11.0)	11 (11.6)
Tibial plateau	60 (35.7)	27 (37.0)	33 (34.7)
**Effusion**			
Retropatellar	61 (36.3)	28 (38.4)	33 (34.7)
Intrapatellar	2 (1.2)	0 (0)	2 (2.1)
**Meniscus lesion**	83 (49.4)	33 (45.2)	50 (52.6)
Lateral	37 (22.0)	17 (23.3)	20 (21.1)
Medial	59 (35.1)	19 (26.0) ^a^	40 (42.1)
**Popliteal Baker’s cyst**	8 (4.8)	4 (5.5)	4 (4.2)
**Patellar subluxation, lateral**	8 (4.8)	2 (2.7)	6 (6.3)
**Patella alta**	30 (17.9)	10 (13.7)	20 (21.1)
**Chondromalacia patellae**	43 (25.6)	17 (23.3)	26 (27.4)
Lateral facet	10 (6.0)	4 (5.5)	6 (6.3)
Central ridge	18 (10.7)	8 (11.0)	10 (10.5)
Medial facet	17 (10.1)	5 (6.8)	12 (12.6)
**Bone bruise**	38 (22.6)	26 (35.6) ^a^	12 (12.6)
Femoral condyle	23 (13.7)	18 (24.7) ^a^	5 (5.3)
Tibial plateau	14 (8.3)	8 (11.0)	6 (6.3)
Patella	5 (3.0)	4 (5.5)	1 (1.1)
**Retinaculum lesion, patella**	19 (11.3)	10 (13.7)	9 (9.5)
**Enthesitis**	42 (25.0)	21 (28.8)	21 (22.1)
M. quadriceps femoral	17 (10.1)	9 (12.3)	8 (8.4)
Patellar tendon	27 (16.1)	12 (16.4)	15 (15.8)
**Fibrosis (cortical, dysplasia, etc.)**	12 (7.1)	7 (9.6)	5 (5.3)
**Ligament injury**	30 (17.9)	21 (28.8) ^a^	9 (9.5)
MCL	8 (4.8)	7 (9.6) ^a^	1 (1.1)
LCL	16 (9.5)	9 (12.3)	7 (7.4)
LCA	12 (7.1)	7 (9.6)	5 (5.3)

^a^ *p* < 0.05 boys vs. girls.

**Table 3:** Age-related differences in prevalence of specific morphological substrates in pediatric patients with knee overuse syndrome.

	Age < 15 years *n* (%)	Age ≥ 15 years *n* (%)
**Cartilage edema**	15 (20.3)	30 (31.9)
Femoral condyle	4 (5.4)	4 (4.3)
Tibial plateau	0 (0.0) ^b^	7 (7.4)
Patella	11 (14.9)	21 (22.3)
**Cartilage rupture**	33 (44.6)	42 (44.7)
Patella	3 (4.1)	7 (7.4)
Femoral condyle	12 (16.2)	7 (7.4)
Tibial plateau	25 (33.8)	35 (37.2)
**Effusion**		
Retropatellar	19 (25.7) ^b^	42 (44.7)
Intrapatellar	2 (2.7)	0 (0)
**Meniscus lesion**	39 (52.7)	44 (46.8)
Lateral	17 (23.0)	20 (21.3)
Medial	27 (36.5)	32 (34.0)
**Popliteal Baker’s cyst**	4 (5.4)	4 (4.3)
**Patellar subluxation, lateral**	4 (5.4)	4 (4.3)
**Patella alta**	14 (18.9)	16 (17.0)
**Chondromalacia patellae**	11 (14.9) ^b^	32 (34.0)
Lateral facet	5 (6.8)	5 (5.3)
Central ridge	2 (2.7) ^b^	16 (17.0)
Medial facet	4 (5.4)	13 (13.8)
**Bone bruise**	15 (20.3)	23 (24.5)
Femoral condyle	9 (12.2)	14 (14.9)
Tibial plateau	6 (8.1)	8 (8.5)
Patella	0 (0)	5 (5.3)
**Retinaculum lesion, patella**	6 (8.1)	13 (13.8)
**Enthesitis**	16 (21.6)	26 (27.7)
M. quadriceps femoral	5 (6.8)	12 (12.8)
Patellar tendon	11 (14.9)	16 (17.0)
**Fibrosis (cortical, dysplasia, etc.)**	8 (10.8)	4 (4.3)
**Ligament injury**	12 (16.2)	18 (19.1)
MCL	3 (4.1)	5 (5.3)
LCL	9 (12.2)	7 (7.4)
LCA	3 (4.1)	9 (9.6)

^b^ *p* < 0.05 age < 15 years vs. age ≥ 15 years.

## Data Availability

The data that support the findings of this study are available on request from the corresponding author.
